# Detection of Puumala Hantavirus Antigen in Human Intestine during Acute Hantavirus Infection

**DOI:** 10.1371/journal.pone.0098397

**Published:** 2014-05-23

**Authors:** Joerg Latus, Klara Tenner-Racz, Paul Racz, Daniel Kitterer, Daniel Cadar, German Ott, M. Dominik Alscher, Jonas Schmidt-Chanasit, Niko Braun

**Affiliations:** 1 Department of Internal Medicine, Division of Nephrology, Robert-Bosch Hospital, Auerbachstrasse 110, Stuttgart, Germany; 2 Bernhard Nocht Institute for Tropical Medicine, WHO Collaborating Centre for Arbovirus and Haemmorrhagic Fever Reference and Research, Bernhard-Nocht-Strasse 74, Hamburg, Germany; 3 Department of Diagnostic Medicine, Division of Pathology, Robert-Bosch Hospital, Auerbachstrasse 110, Stuttgart, Germany; 4 German Centre for Infection Research (DZIF), partner site Hamburg-Luebeck-Borstel, Hamburg, Germany; Karolinska Institutet, Sweden

## Abstract

**Background:**

Puumala virus (PUUV) is the most important hantavirus species in Central Europe. Nephropathia epidemica (NE), caused by PUUV, is characterized by acute renal injury (AKI) with thrombocytopenia and frequently gastrointestinal symptoms.

**Methods:**

456 patients with serologically and clinically confirmed NE were investigated at time of follow-up in a single clinic. The course of the NE was investigated using medical reports. We identified patients who had endoscopy with intestinal biopsy during acute phase of NE. Histopathological, immunohistochemical and molecular analyses of the biopsies were performed.

**Results:**

Thirteen patients underwent colonoscopy or gastroscopy for abdominal pain, diarrhea, nausea and vomiting during acute phase of NE. Immunohistochemistry (IHC) revealed PUUV nucleocapsid antigen in 11 biopsies from 8 patients; 14 biopsies from 5 patients were negative for PUUV nucleocapsid antigen. IHC localized PUUV nucleocapsid antigen in endothelial cells of capillaries or larger vessels in the lamina propria. Rate of AKI was not higher and severity of AKI was not different in the PUUV-positive compared to the PUUV-negative group. All IHC positive biopsies were positive for PUUV RNA using RT-PCR. Phylogenetic reconstruction revealed clustering of all PUUV strains from this study with viruses previously detected from the South-West of Germany. Long-term outcome was favorable in both groups.

**Conclusions:**

In patients with NE, PUUV nucleocapsid antigen and PUUV RNA was detected frequently in the intestine. This finding could explain frequent GI-symptoms in NE patients, thus demonstration of a more generalized PUUV infection. The RT-PCR was an effective and sensitive method to detect PUUV RNA in FFPE tissues. Therefore, it can be used as a diagnostic and phylogenetic approach also for archival materials. AKI was not more often present in patients with PUUV-positive IHC. This last finding should be investigated in larger numbers of patients with PUUV infection.

## Introduction

Hantaviruses are rodent-borne zoonotic viruses of the *Bunyaviridae* family [1] that can cause Hemorrhagic Fever with Renal Syndrome (HFRS) in Asia and Europe and Hantavirus Cardiopulmonary Syndrome in the Americas with reported case fatality rates up to 35% [2]–[5]. Puumala virus (PUUV) is the main hantavirus species in Central Europe and is known to cause a milder form of HFRS, which also is called nephropathia epidemica (NE) [6]–[11]. The clinical picture in NE includes acute kidney injury (AKI), thrombocytopenia, proteinuria and hematuria caused by tubular and glomerular involvement [12]. Both renal tubular and glomerular cells are affected by infection that leads to breakdown of cell-to cell contacts [13]. Therefore, the urinary sediment of patients with hantavirus infection contains tubular cells, with enlarged nucleoli, that are positive for hantavirus antigen [14]–[16]. At the level of histopathology, hantavirus infection leads to mild tubular interstitial changes and moderate interstitial infiltration with mononuclear cells [13], [17]. Up to now, hantavirus antigen has been found only in the cytoplasm of tubular epithelial cells from kidney biopsies of infected patients [16], [18]–[21]. In 1992, Nuutinen et al. [22] were the first who performed endoscopy (gastroscopy or colonoscopy) in 10 patients with acute NE (within 1–4 weeks after beginning of symptoms). In every case, hemorrhagic gastropathy was observed. Histopathological analysis of the intestinal biopsies showed that the hemorrhagic lesions were associated with edema in the lamina propria. No inflammatory changes could be detected within the biopsies [22].

A high percentage of patients with PUUV infection have severe gastrointestinal (GI) symptoms, e.g. abdominal pain, nausea and vomiting [12], [23], [24]. Recently we demonstrated PUUV antigen in the appendix of a NE patient with severe AKI, who underwent surgery due to severe abdominal pain. Immunohistochemistry (IHC) of the appendix from this patient showed PUUV antigen in capillary and large vessel endothelium. The capillaries with PUUV-positive, endothelial cells were located mainly in the lamina propria, including the gut associated lymphoid tissue (GALT). PUUV antigen in larger vessels was present mainly in the serosa and mesoappendix [25]. Rarely, viral antigen was found in lymphoreticular cells. In the present study, our aim was to investigate whether GI symptoms in some patients with NE is associated with PUUV infection of intestine. We have performed histopathological, immunohistochemical and molecular analyses of biopsies of the human intestine of 13 patients with NE and severe GI symptoms. The course of the disease and long- term outcome was investigated in these patients.

## Materials and Methods

### Patients

Since 2001, German laboratories report diagnosed PUUV infections to the local health authorities. All serologically and clinically confirmed NE patients from four selected local health authorities in southern Germany were contacted by mail. We identified 1.570 patients with NE between who became ill between 2001 and 2012. These patients were asked to attend the outpatient clinic between September 2012 and April 2013. Overall 456 NE patients could be followed at the outpatient department at the Robert-Bosch-Hospital Stuttgart, Germany. Three patients were excluded by age <18 years at the time of NE. All patients gave written informed consent before participating in the study, which was approved by the Ethics Committee of the Ethics Commission of the State Chamber of Medicine in Baden- Wuerttemberg (Stuttgart) (F-2012-046). Studies were conducted in concordance with the Declaration of Helsinki.

### Data acquisition

Clinical and laboratory data during the acute phase of NE were obtained from medical reports and files from each patient or reported by the patient at time of follow-up. We identified patients who underwent colonoscopy or gastroscopy including biopsies during acute PUUV- infection. At time of follow-up at the outpatient department at the Robert-Bosch-Hospital Stuttgart, Germany detailed past and current medical histories were obtained, and a careful physical examination was done. AKI was classified on the basis of the RIFLE criteria [26]. Mild/moderate AKI was defined in patients with no AKI and AKI Risk (R) and severe AKI was classified as RIFLE Injury (I) and RIFLE Failure (F). Information on pre-hospital baseline serum creatinine levels was available for all patients. Blood pressure was measured in all patients twice as recommended by the American Heart Association [27]. Hypertension stage 1 was defined by SBP of 140–159 mm Hg or DBP 90–99 mmHg and hypertension stage 2 by SBP >160 mm Hg or DBP >100 mmHg according to the classification of blood pressure for adults of the Seventh Report of the Joint National Committee on Prevention, Detection, Evaluation, and Treatment of High Blood Pressure [28].

All serum samples at time of follow-up were analyzed for PUUV-specific IgM and IgG in all patients using a strip-immunoblotassay (recomLine Bunyavirus IgG/IgM, Mikrogen, Germany) [29]. Hematuria was defined as a positive dipstick test for erythrocytes and over two erythrocytes per high-power field. Proteinuria was defined by an albumin/creatinine ratio (ACR)>0.25 g/g creatinine in spot urine sample [30].

### Biopsies and IHC

All patients had given their informed consent regarding a scientific work-up of tissues taken during routine procedures and eight different departments of pathology were involved in the routine pathological work-up of the biopsies. Twenty-five intestinal biopsies were fixed in 10% neutral-buffered formalin solution and embedded in paraffin. Five µm-thick sections were prepared and placed on slides coated with 3-amino-propyl-trethosilane. After deparaffinizing through xylene the sections were boiled in a domestic pressure cooker in citrate buffer pH 6 for 3 minutes and chilled down to room temperature. The sections were incubated with a PUUV monoclonal antibody raised against nucleocapsid protein (clone A1C5; dilution: 1∶30; Progen Biotechnik, Heidelberg, Germany) for 30 min. Binding of antibody was visualized using the kit DCS Detection Line (DCS Innovative Diagnostik System, Hamburg, Germany) containing a secondary antibody coupled with biotin and a conjugate Streptavidin and horseradish peroxidase. The slides were developed using DAB (3,3′diaminobenzidine), counterstained with haematoxylin and mounted. To delineate the cells containing PUUV nucleocapsidantigen, immunohistochemical double labelling was applied. After developing the DAB step, the sections were then heat treated again for 2 min and incubated with the monoclonal antibody CD34 (dilution: 1∶100; Dako) for overnight. The second antibody was visualized with alkaline phosphatase (Dako) Fast Blue salt. The sections were examined using an AxioIimager M1 microscope (Carl Zeiss, Jena, Germany). Vero E6 cells infected with PUUV strain Sotkamo served as positive control.

### Molecular detection and phylogenetic characterization of PUUV from biopsies

A total of 8 biopsies from different patients (7 gastric and 1 colon), which were detected as positive by IHC, have been subjected to molecular investigation. In parallel, we used formalin fixed and paraffin embedded (FFPE) PUUV uninfected and infected Vero E6 cells as controls. Four 10-micron thickness sections from each FFPE biopsies were used for RNA extraction. Whole viral RNA was extracted from tissues by use of RNeasy FFPE Kit (Qiagen, Hilden, Germany) according to the manufacturer's instructions. RNA was then subjected to RT-PCR to recover partial S segment (144 bp) using a degenerate forward primer PUMAGJ1-F 5′-CAACMCGTGGGAGACARACTGT-3′ at position 429 and reverse primer PUMAGJ1-R 5′-CYTTCATRGTTGAYTGAGCAGT-3′ at position 572 both positions according to the complete nucleocapsid gene protein sequence of the Bavaria CG 33/04 strain (GenBank acc. no. DQ016430) designed based on all available European PUUV strains retrieved from the GenBank. Reverse transcription and PCR amplification (RT-PCR) of the 144 bp segment of the nucleocapsid gene was performed using the SuperScript III One-Step RT-PCR System with Platinum Taq High Fidelity (Invitrogen). The PCR was performed in a 50-ml volume containing 5 µl of sample RNA, 25 µl of 2x Reaction Mix, 2.5 µl of each primer (PUMAGJ1 and PUMAGJ2), 2 µl High Fidelity Enzym-Mix and ddH2O up to 50 µl. The reaction was then incubated in a thermocycler under the following conditions: 50°C for 50 minutes and 94°C as cDNA synthesis and pre-denaturation step; 45 cycles of 94°C for 20 seconds (denaturation), 55°C for 45 seconds (annealing), 72°C for 1 minute (extension) with a final elongation step at 72 °C for 10 min. The PCR product was visualized on a UV transilluminator following separation on 2.5% agarose gels containing ethidium bromide. Amplicons were sent to the company LGC Genomics for Sanger sequencing. Sequence data were analyzed using Geneious 7.4.1 program for multiple alignments. Phylogenetic analyses of the obtained sequences were performed in comparison to the strains belonging to different geographical regions as retrieved from GenBank. Evolutionary tree and distances (number of base substitutions per site) were generated by PhyML v3.1 [31]with the General-Time-Reversible model of sequence evolution with gamma-distributed rate variation among sites and proportion of invariable sites (GTR +Γ+I) found to be most appropriate model according to Akaike information criteria included in the jModelTest [32]. The PUUV sequences were shown in File S1.

### Statistical analysis

Data are reported as median and range unless otherwise specified. All continuous variables were tested for normality using the Kolmogorov-Smirnov test. Comparisons between different groups were performed with the Mann-Whitney test or the Fisher's exact test, as appropriate. Analysis was performed using GraphPad statistical software package (San Diego, California, USA). A p value <0.05 was considered statistically significant.

## Results

Overall, 456 patients were followed-up at our outpatient clinic with a median follow up time of 10 (7–29) months after acute NE. All patients lived in southern Germany and had laboratory and clinically confirmed NE between 2001 and 2012.Thirteen patients had colonoscopy or gastroscopy with biopsies during their acute PUUV- infection. Endoscopy was done 12 (9–15) days in the PUUV-positive group and 7 (4–9) days in the PUUV-negative group after beginning of symptoms associated with NE. The indications for endoscopy were severe abdominal pain, diarrhea, nausea and vomiting. Gastroscopy revealed gastritis with subepithelial hemorrhages and telangiectasia in all patients. The most prominent hemorrhagic findings were found in the antrum. In the PUUV-positive group, in two patients gastritis was associated with *Helicobater pylori* infection, whereas in the PUUV-negative group no *Helicobater pylori* infection could be found. In the PUUV-negative group, two patients had mild gastro-esophageal reflux disease and in one patient thrush esophagitis was present. Colonoscopy was done in 3 patients revealing spotty hemorrhage in all biopsies without any signs of inflammation.

**Figure 1:**
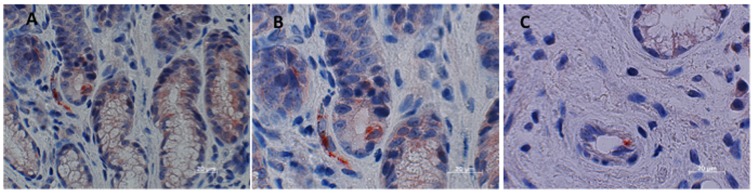
Detection of PUUV nucleocapsid antigen in the capillaries in the lamina propria of a gastric biopsy 8 days after beginning of symptoms associated with NE (fever, abdominal pain, nausea and vomiting) A Higher magnification demonstrates capillaries with positive endothelial cells in the lamina propria (same patient) B Endothelial cell with PUUV nucleocapsid antigen (same patient) C.

**Figure 2:**
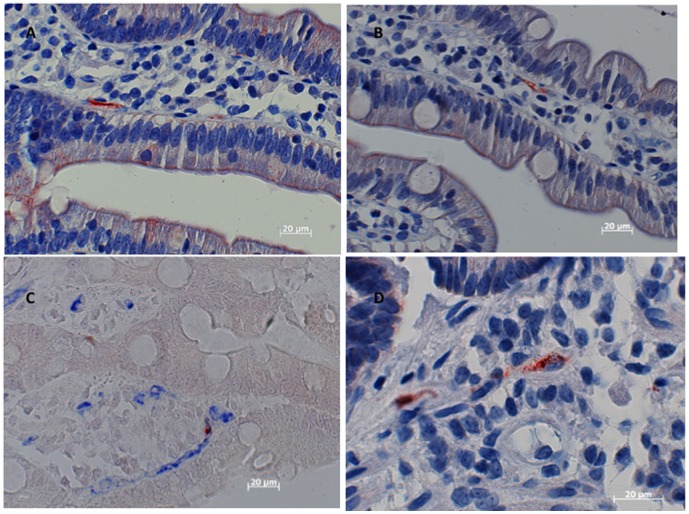
Detection of PUUV nucleocapsid antigen in the capillaries in the lamina propria (gastric biopsy) 10 days after beginning of symptoms A and B Immunohistochemical double labeling with the monoclonal antibody A1C5 (brown) and the monoclonal antibody to CD34 reacting the endothelial cells (blue). The PUUV nucleocapsid antigen is granular C Detection of PUUV nucleocapsid antigen in interstitial cells (gastric biopsy) D.

IHC was done on 25 intestinal biopsies. Twenty-one of these were gastric; 1 was esophageal; and 3 were from colon. PUUV nucleocapsid antigen was detected in eleven biopsies from eight patients (9 gastric and 2 colon). Fourteen biopsies (12 gastric, 1 esophageal and 1 colon) from 5 patients were negative for PUUV nucleocapsid antigen. PUUV nucleocapsid antigen was mainly found in endothelial cell of capillaries or larger vessels in the lamina propria of biopsies from stomach or colon (see Figure 1 and 2). The intense immunohistochemical staining of cells expressing PUUV nucleocapsid antigen deposited in a granular pattern documents the involvement of the human intestine in PUUV infection. Biopsies from all patients, which were detected as positive by IHC, have been subjected to molecular investigation. All IHC positive biopsies were positive for PUUV RNA using RT-PCR.

Four out of eight patients in the PUUV-positive and three out of five patients in the PUUV-negative group presented with abdominal pain at time of diagnosis. Nausea and vomiting were present in five out of eight patients in the PUUV-positive and in three out of five patients in the PUUV negative group. In total, ten out of thirteen patients had AKI during PUUV infection. Neither higher rates of AKI, nor differences regarding severity of AKI could be detected in the PUUV-positive compared to the PUUV- negative group. At time of follow-up, kidney function was within the normal range in all patients. The prevalence of hypertension, proteinuria, hematuria and co-morbidities as long-term consequences after PUUV infection was not different between both groups. A detailed summary of study population including laboratory values during acute course of the disease and at time of follow-up is given in table 1 and table 2. Due to a possible selection bias, we compared baseline characteristics during acute course of the disease (e.g. laboratory values, severity of AKI, symptoms during acute course of the disease) and outcome data between the patients who underwent endoscopy with biopsies (n = 13) and the study population without endoscopy (n = 443 patients). No statistical significant differences could be observed between the groups (data not shown).

**Table 1:** Baseline characteristics of study population during acute phase of NE.

Variable	PUUV nucleocapsid antigen positive (n = 8)	PUUV nucleocapsid antigen negative (n = 5)
n	8	5
Female/male	4/4	1/4
Age at diagnosis (years)	55 (48–65)	51 (38–63)
In-patients/outpatients	8/0	4/1
Duration of hospital stay	9 (7–13)	11 (9–12)
Assumed path of infection		
- Occupational	0	1
- During leisure time	8	4
**Symptoms**		
Abdominal pain	4/8	3/5
Back pain	3/8	4/5
Pain of the limbs	6/8	3/5
Headache	6/8	4/5
Visual disorders	1/8	0/5
Diarrhea	2/8	2/5
Nausea/Vomiting	5/8	3/5
**Clinical signs**		
Fever	7/8	5/5
Hemorrhage	0/8	0/5
**Urinary analysis at admission**	6/8	4/4
- Proteinuria	5/6	3/5
- Hematuria	4/6	1/5
- Leucocyturia	1/6	0/5
**Acute Kidney Injury**		
Acute Kidney Injury according to RIFLE classification	7/8	3/5
- Risk	5	1
- Injury	0	1
- Failure	2	1

**Table 2:** Baseline characteristics of study population at follow-up.

Variable	PUUV nucleocapsid antigen positive (n = 8)	PUUV nucleocapsid antigen negative (n = 5)
Number of patients	8	5
Follow-up (months)	10 (7–33)	19 (10–28)
**Laboratory findings**		
Creatinine (mg/dl [0.5–1.4])	0.9 (0.8–1.0)	0.9 (0.8–0.9)
Platelets (10^9^/L [>150])	238 (220–256)	241 (216–258)
**Urinary analysis**		
- Proteinuria	0/8	0/5
- Hematuria	2/8	0/5
- Leucocyturia	2/8	0/5
- Presence of bacteria	0/8	0/5
**Hypertension**		
Hypertension at time of presentation	1/8	1/5

**Figure 3:**
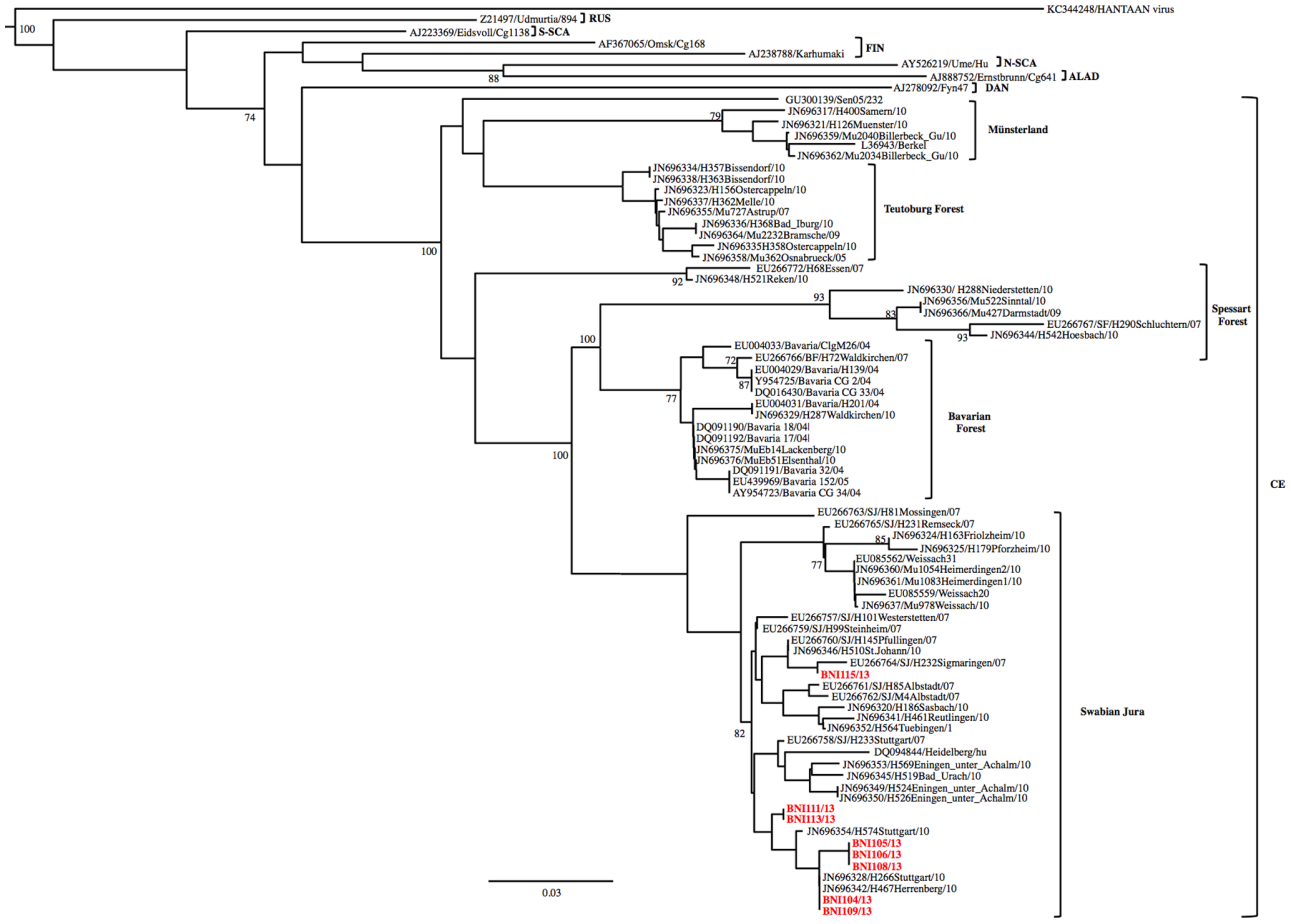
Maximum-likelihood phylogenetic tree of PUUV based on partial (144 bp) sequences of the small segment showing the placement of the PUUV strains from this study into the German subtypes. Bootstrap values >70%, calculated from 10,000 replicates are shown at the tree branches. For clarity, the Central European PUUMV clade include only German strains. Previously characterized PUUMV clades: **ALAD**, Alpe-Adrian; **CE**, Central European; **S-SCA**, South Scandinavian; **FIN**, Finnish; **RUS**, Russian; **N-SCA**, North Scandinavian; **DAN**, Dannish. The tree was rooted using murine-associated hantavirus: Hantaan virus (HNTV). Scale bar indicates mean number of nucleotide substitutions per site. GenBank accession numbers and the name of the strains are shown.

Phylogenetic reconstruction revealed clustering of all PUUV strains from this study with viruses previously detected from the South-West of Germany (Figure 3) [33].

## Discussion

We investigated biopsies of the human intestine in patients with acute PUUV infection in order to understand why a majority of patients with NE have severe abdominal pain [23], for which there is no pathophysiologic explanation. We found 13 patients who had an endoscopy at time of acute Hantavirus infection due to severe gastrointestinal symptoms. Endoscopy was carried out during acute phase of NE and gastritis with hemorrhage could be observed in all patients. In line with the results from to the study of Nuutinen et al. [22], the hemorrhagic changes were more prominent in the proximal compared to the distal part of the stomach. In contrast, signs of inflammation could be detected in all intestinal biopsies in our study.

IHC demonstrated PUUV nucleocapsid antigen in 62% of the analyzed biopsies. PUUV nucleocapsid antigen was located mainly in endothelial cells of capillaries or larger vessels in the lamina propria. This finding accords with previous reports from patients with HCPS, with experimental PUUV infection in cynomolgus macaques and from our recent report [25], [34], [35]. Almost all hantavirus infections (especially southern Germany) are caused by PUUV [7], [33], although Tula virus and Dobrava-Belgrade virus circulates in Germany and might cause symptomatic infections in humans [36]–[39].

Regarding clinical course of the disease, twelve out of thirteen patients had to be admitted to hospital. Duration of hospital stay was more than one week and ten out of thirteen patients had AKI, indicating that PUUV causes severe disease in most patients with NE. Although the number of patients is relatively small, a possible explanation could be an enhanced inflammatory response caused by a generalized PUUV infection. This systemic infection could be reflected by the presence of PUUV nucleocapsid antigen in human intestine and might explain the clinical phenotype of severe GI-symptoms. Further studies in larger numbers of NE patients are needed to investigate whether detection of PUUV nucleocapsid antigen in the human intestine might be a risk factor for severe course of the disease.

Remarkably, all IHC positive biopsies were positive for PUUV RNA using RT-PCR. Thus, the RT-PCR technique could be used to detect infection from archival or fixed materials. As an auxiliary advantage of RT-PCR, it is possible to sequence the amplicon, enabling more extensive molecular analyses. In this study, the nucleocapsid gene was chosen for amplification because it provides valuable information regarding the possible origin of the PUUV. The success of the development of this RT-PCR from FFPE biopsies enables work on PUUV in more flexible conditions because FFPE could be handled without biosecurity concerns. In this study, phylogenetic reconstruction revealed clustering of all PUUV strains with viruses previously detected from South-West of Germany [33]. Based on Ettinger et al. [33] the German PUUV strains formed 6 distinct clades comprising strains of human and vole origin corresponding to a different outbreak region. High similarities between human and rodent-derived PUUV strains from the same region and distinct from the others highlight the fact that the 2010 epidemic was associated with increased densities and infection rates of the bank voles in several regions of Germany and not determined by countrywide spread of the same PUUV strain [33]. In conclusion, we successfully developed a method to detect PUUV in FFPE tissues by RT-PCR and demonstrated its use as potential diagnostic tool as well as for PUUV molecular epidemiology investigations. Furthermore, it is possible to perform retrospective studies of archived materials from patients where PUUV was not the initial diagnosis.

This is the first study, which investigated biopsies of the human intestine in patients with NE using IHC and molecular approaches. PUUV nucleocapsid antigen and PUUV RNA were frequently detected. This finding might be an expression of a more generalized infection, which is emphasized by high rates of AKI in this study population. The presence of PUUV antigen was not correlated with the extension of abdominal complains. We suggest that a larger patient cohort should be analyzed in future studies to demonstrate possible significant differences and correlations.

## Supporting Information

File S1 **PUUV sequences.**
(DOC)

## References

[1] Schmaljohn CS, Dalrymple JM (1983) Analysis of Hantaan virus RNA: evidence for a new genus of bunyaviridae. Virology 131: 482–491. 6419460 10.1016/0042-6822(83)90514-7

[2] Kruger DH, Schonrich G, Klempa B (2011) Human pathogenic hantaviruses and prevention of infection. Hum Vaccin 7: 685–693. 21508676 10.4161/hv.7.6.15197 PMC3219076

[3] Lee HW, van der Groen G (1989) Hemorrhagic fever with renal syndrome. Prog Med Virol 36: 62–102. 2573914

[4] Mertz GJ, Hjelle B, Crowley M, Iwamoto G, Tomicic V, et al (2006) Diagnosis and treatment of new world hantavirus infections. Curr Opin Infect Dis 19: 437–442. 16940866 10.1097/01.qco.0000244048.38758.1f

[5] Peters CJ, Simpson GL, Levy H (1999) Spectrum of hantavirus infection: hemorrhagic fever with renal syndrome and hantavirus pulmonary syndrome. Annu Rev Med 50: 531–545. 10073292 10.1146/annurev.med.50.1.531

[6] Vapalahti O, Mustonen J, Lundkvist A, Henttonen H, Plyusnin A, et al (2003) Hantavirus infections in Europe. Lancet Infect Dis 3: 653–661. 14522264 10.1016/s1473-3099(03)00774-6

[7] Kruger DH, Ulrich RG, Hofmann J (2013) Hantaviruses as zoonotic pathogens in Germany. Dtsch Arztebl Int 110: 461–467. 23964302 10.3238/arztebl.2013.0461 PMC3722641

[8] Hofmann J, Meisel H, Klempa B, Vesenbeckh SM, Beck R, et al (2008) Hantavirus outbreak, Germany, 2007. Emerg Infect Dis 14: 850–852. 18439382 10.3201/eid1405.071533 PMC2600236

[9] Heyman P, Thoma BR, Marie JL, Cochez C, Essbauer SS (2012) In Search for Factors that Drive Hantavirus Epidemics. Front Physiol 3: 237. 22934002 10.3389/fphys.2012.00237 PMC3429022

[10] Mertens M, Wolfel R, Ullrich K, Yoshimatsu K, Blumhardt J, et al (2009) Seroepidemiological study in a Puumala virus outbreak area in South-East Germany. Med Microbiol Immunol 198: 83–91. 19148676 10.1007/s00430-009-0106-9

[11] Essbauer SS, Schmidt-Chanasit J, Madeja EL, Wegener W, Friedrich R, et al (2007) Nephropathia epidemica in metropolitan area, Germany. Emerg Infect Dis 13: 1271–1273. 17953116 10.3201/eid1308.061425 PMC2828076

[12] Krautkramer E, Zeier M, Plyusnin A (2013) Hantavirus infection: an emerging infectious disease causing acute renal failure. Kidney Int 83: 23–27. 23151954 10.1038/ki.2012.360

[13] Krautkramer E, Grouls S, Stein N, Reiser J, Zeier M (2011) Pathogenic old world hantaviruses infect renal glomerular and tubular cells and induce disassembling of cell-to-cell contacts. J Virol 85: 9811–9823. 21775443 10.1128/JVI.00568-11 PMC3196447

[14] Zeier M, Ritz E (1996) [Hantavirus-induced acute renal failure]. Internist (Berl) 37: 1092–1095. 9036104

[15] Mustonen J, Helin H, Pietila K, Brummer-Korvenkontio M, Hedman K, et al (1994) Renal biopsy findings and clinicopathologic correlations in nephropathia epidemica. Clin Nephrol 41: 121–126. 7910539

[16] Groen J, Bruijn JA, Gerding MN, Jordans JG, Moll van Charante AW, et al (1996) Hantavirus antigen detection in kidney biopsies from patients with nephropathia epidemica. Clin Nephrol 46: 379–383. 8982553

[17] Muranyi W, Bahr U, Zeier M, van der Woude FJ (2005) Hantavirus infection. J Am Soc Nephrol 16: 3669–3679. 16267154 10.1681/ASN.2005050561

[18] Hautala T, Sironen T, Vapalahti O, Paakko E, Sarkioja T, et al (2002) Hypophyseal hemorrhage and panhypopituitarism during Puumala Virus Infection: Magnetic Resonance Imaging and detection of viral antigen in the hypophysis. Clin Infect Dis 35: 96–101. 12060884 10.1086/340859

[19] Hung T, Zhou JY, Tang YM, Zhao TX, Baek LJ, et al (1992) Identification of Hantaan virus-related structures in kidneys of cadavers with haemorrhagic fever with renal syndrome. Arch Virol 122: 187–199. 1346088 10.1007/BF01321127

[20] Kim S, Kang ET, Kim YG, Han JS, Lee JS, et al (1993) Localization of Hantaan viral envelope glycoproteins by monoclonal antibodies in renal tissues from patients with Korean hemorrhagic fever H. Am J Clin Pathol 100: 398–403. 7692720 10.1093/ajcp/100.4.398

[21] Temonen M, Mustonen J, Helin H, Pasternack A, Vaheri A, et al (1996) Cytokines, adhesion molecules, and cellular infiltration in nephropathia epidemica kidneys: an immunohistochemical study. Clin Immunol Immunopathol 78: 47–55. 8599883 10.1006/clin.1996.0007

[22] Nuutinen H, Vuoristo M, Farkkila M, Kahri A, Seppala K, et al (1992) Hemorrhagic gastropathy in epidemic nephropathy. Gastrointest Endosc 38: 476–480. 1355053 10.1016/s0016-5107(92)70480-5

[23] Braun N, Haap M, Overkamp D, Kimmel M, Alscher MD, et al (2010) Characterization and outcome following Puumala virus infection: a retrospective analysis of 75 cases. Nephrol Dial Transplant 25: 2997–3003. 20223893 10.1093/ndt/gfq118

[24] Vaheri A, Strandin T, Hepojoki J, Sironen T, Henttonen H, et al (2013) Uncovering the mysteries of hantavirus infections. Nat Rev Microbiol 11: 539–550. 24020072 10.1038/nrmicro3066

[25] Latus J, Fritzenkotter M, Schmidt-Chanasit J, Tenner-Racz K, Leibold T, et al (2012) Hantavirus and acute appendicitis—the diagnosis behind the diagnosis? J Clin Virol 53: 156–158. 22169597 10.1016/j.jcv.2011.11.003

[26] Bellomo R, Ronco C, Kellum JA, Mehta RL, Palevsky P (2004) Acute renal failure - definition, outcome measures, animal models, fluid therapy and information technology needs: the Second International Consensus Conference of the Acute Dialysis Quality Initiative (ADQI) Group. Crit Care 8: R204–212. 15312219 10.1186/cc2872 PMC522841

[27] Pickering TG, Hall JE, Appel LJ, Falkner BE, Graves J, et al (2005) Recommendations for blood pressure measurement in humans and experimental animals: Part 1: blood pressure measurement in humans: a statement for professionals from the Subcommittee of Professional and Public Education of the American Heart Association Council on High Blood Pressure Research. Hypertension 45: 142–161. 15611362 10.1161/01.HYP.0000150859.47929.8e

[28] Chobanian AV, Bakris GL, Black HR, Cushman WC, Green LA, et al (2003) The Seventh Report of the Joint National Committee on Prevention, Detection, Evaluation, and Treatment of High Blood Pressure: the JNC 7 report. JAMA 289: 2560–2572. 12748199 10.1001/jama.289.19.2560

[29] Zoller LG, Yang S, Gott P, Bautz EK, Darai G (1993) A novel mu-capture enzyme-linked immunosorbent assay based on recombinant proteins for sensitive and specific diagnosis of hemorrhagic fever with renal syndrome. J Clin Microbiol 31: 1194–1199. 8099085 10.1128/jcm.31.5.1194-1199.1993 PMC262902

[30] Schwab SJ, Christensen RL, Dougherty K, Klahr S (1987) Quantitation of proteinuria by the use of protein-to-creatinine ratios in single urine samples. Arch Intern Med 147: 943–944. 3555378

[31] Guindon S, Dufayard JF, Lefort V, Anisimova M, Hordijk W, et al (2010) New algorithms and methods to estimate maximum-likelihood phylogenies: assessing the performance of PhyML 3.0. Syst Biol 59: 307–321. 20525638 10.1093/sysbio/syq010

[32] Darriba D, Taboada GL, Doallo R, Posada D (2012) jModelTest 2: more models, new heuristics and parallel computing. Nat Methods 9: 772. 10.1038/nmeth.2109 PMC4594756 22847109

[33] Ettinger J, Hofmann J, Enders M, Tewald F, Oehme RM, et al (2012) Multiple synchronous outbreaks of Puumala virus, Germany, 2010. Emerg Infect Dis 18: 1461–1464. 22932394 10.3201/eid1809.111447 PMC3437711

[34] Clement J, Maes P, van Ypersele de Strihou C, van der Groen G, Barrios JM, et al (2010) Beechnuts and outbreaks of nephropathia epidemica (NE): of mast, mice and men. Nephrol Dial Transplant 25: 1740–1746. 20237057 10.1093/ndt/gfq122

[35] Sironen T, Klingstrom J, Vaheri A, Andersson LC, Lundkvist A, et al (2008) Pathology of Puumala hantavirus infection in macaques. PLoS One 3: e3035. 18716663 10.1371/journal.pone.0003035 PMC2516326

[36] Maes P, Clement J, Gavrilovskaya I, Van Ranst M (2004) Hantaviruses: immunology, treatment, and prevention. Viral Immunol 17: 481–497. 15671746 10.1089/vim.2004.17.481

[37] Klempa B, Meisel H, Rath S, Bartel J, Ulrich R, et al (2003) Occurrence of renal and pulmonary syndrome in a region of northeast Germany where Tula hantavirus circulates. J Clin Microbiol 41: 4894–4897. 14532254 10.1128/JCM.41.10.4894-4897.2003 PMC254384

[38] Klempa B, Schutt M, Auste B, Labuda M, Ulrich R, et al (2004) First molecular identification of human Dobrava virus infection in central Europe. J Clin Microbiol 42: 1322–1325. 15004109 10.1128/JCM.42.3.1322-1325.2004 PMC356881

[39] Ulrich RG, Imholt C, Kruger DH, Krautkramer E, Scheibe T, et al (2013) [Hantaviruses in Germany: threat for zoo, pet, companion and farm animals?]. Berl Munch Tierarztl Wochenschr 126: 514–526. 24511827

