# Supplementary material for: Detection of Puumala Hantavirus Antigen in Human Intestine during Acute Hantavirus Infection
Source: PLoS One. 2014 May 23;9(5):e98397. doi: 10.1371/journal.pone.0098397 (PMC4032337; doi:10.1371/journal.pone.0098397)
Supplement: File S1 — PUUV sequences. (DOC) [file pone.0098397.s001.doc]

[Puumala virus strain 104-13 ] nucleocapsid protein, partial cds.

CAACCCGTGGGAGACAGACTGTGAAGGAAAACAAAGGGACTCGTATTCGGTTTAAAGATGATACTTCTTTCGAGGACATTAATGGTATTAGAAGGCCAAAGCACTTGTATGTGTCCATGCCGACTGCTCAATCAACCATGAAAG

[Puumala virus strain 105-13 ] nucleocapsid protein, partial cds.

CAACCCGTGGGAGACAAACTGTGAAGGAAAACAAAGGGACTCGTATTCGGTTTAAAGATGATACTTCTTTCGAGGACATTAATGGTATTAGAAGGCCAAAGCACTTGTATGTGTCCATGCCGACTGCTCAATCAACCATGAAAG

[Puumala virus strain 106-13 ] nucleocapsid protein, partial cds.

CAACCCGTGGGAGACAAACTGTGAAGGAAAACAAAGGGACTCGTATTCGGTTTAAAGATGATACTTCTTTCGAGGACATTAATGGTATTAGAAGGCCAAAGCACTTGTATGTGTCCATGCCGACTGCTCAATCAACCATGAAAG

[Puumala virus strain 108-13 ] nucleocapsid protein, partial cds.

CAACCCGTGGGAGACAAACTGTGAAGGAAAACAAAGGGACTCGTATTCGGTTTAAAGATGATACTTCTTTCGAGGACATTAATGGTATTAGAAGGCCAAAGCACTTGTATGTGTCCATGCCGACTGCTCAATCAACCATGAAAG

[Puumala virus strain 109-13 ] nucleocapsid protein, partial cds.

CAACCCGTGGGAGACAGACTGTGAAGGAAAACAAAGGGACTCGTATTCGGTTTAAAGATGATACTTCTTTCGAGGACATTAATGGTATTAGAAGGCCAAAGCACTTGTATGTGTCCATGCCGACTGCTCAATCAACCATGAAAG

[Puumala virus strain 111-13 ] nucleocapsid protein, partial cds.

CAACCCGTGGGAGACAGACTGTGAAGGAAAACAAAGGGACTCGTATTCGGTTTAAAGATGATACTTCTTTCGAGGACATTAATGGTATTAGAAGACCAAAGCACTTGTATGTGTCCATGCCAACTGCTCAATCAACCATGAAAG

[Puumala virus strain 113-13 ] nucleocapsid protein, partial cds.

CAACCCGTGGGAGACAGACTGTGAAGGAAAACAAAGGGACTCGTATTCGGTTTAAAGATGATACTTCTTTCGAGGACATTAATGGTATTAGAAGACCAAAGCACTTGTATGTGTCCATGCCAACTGCTCAATCAACCATGAAAG

[Puumala virus strain 115-13 ] nucleocapsid protein, partial cds.

CAACCCGTGGGAGACAGACTGTGAAGGAAAACAAAGGGACTCGTATTCGGTTTAAAGATGATACTTCTTTTGAGGACATTAATGGTATTAGAAGACCAAAGCATTTGTATGTGTCTATGCCGACTGCTCAATCAACCATGAAAG
